# Thermal comfort in urban green spaces: a survey on a Dutch university campus

**DOI:** 10.1007/s00484-016-1193-0

**Published:** 2016-06-20

**Authors:** Yafei Wang, Rudolf de Groot, Frank Bakker, Heinrich Wörtche, Rik Leemans

**Affiliations:** 1 Environmental System Analysis Group, Wageningen University, P.O. Box 47, 6700 AA Wageningen, The Netherlands; 2 INCAS3, P.O. Box 797, 9400 AT Assen, The Netherlands

**Keywords:** Outdoor thermal comfort ∙ Urban green infrastructure ∙ Thermal adaptation ∙ Temperate regions ∙ Correlation analysis

## Abstract

To better understand the influence of urban green infrastructure (UGI) on outdoor human thermal comfort, a survey and physical measurements were performed at the campus of the University of Groningen, The Netherlands, in spring and summer 2015. Three hundred eighty-nine respondents were interviewed in five different green spaces. We aimed to analyze people’s thermal comfort perception and preference in outdoor urban green spaces, and to specify the combined effects between the thermal environmental and personal factors. The results imply that non-physical environmental and subjective factors (e.g., natural view, quiet environment, and emotional background) were more important in perceiving comfort than the actual thermal conditions. By applying a linear regression and probit analysis, the comfort temperature was found to be 22.2 °C and the preferred temperature was at a surprisingly high 35.7 °C. This can be explained by the observation that most respondents, who live in temperate regions, have a natural tendency to describe their preferred state as “warmer” even when feeling “warm” already. Using the Kruskal-Wallis *H* test, the four significant factors influencing thermal comfort were people’s exposure time in green spaces, previous thermal environment and activity, and their thermal history. However, the effect of thermal history needs further investigation due to the unequal sample sizes of respondents from different climate regions. By providing evidence for the role of the objective and subjective factors on human thermal comfort, the relationship between UGI, microclimate, and thermal comfort can assist urban planning to make better use of green spaces for microclimate regulation.

## Introduction

The accelerated population growth in urban areas, associated with the increase of impermeable concrete surfaces, industrial pollution, and destruction of natural habitats, negatively changes the urban microclimate (Watson and Johnson 1987; Akbari et al. 2001; Grimmond 2007). The impacts of these changes on microclimate and human thermal comfort have negative effects on human health and received increasing attention (Campbell-Lendrum and Corvalán 2007; Zhao et al. 2011; Franck et al. 2013). In addition, interest in the effects of urban green infrastructure (UGI) on thermal perception and microclimate is growing (Hwang et al. 2010; Krüger et al. 2011; Lin et al. 2013; Yang et al. 2013). Besides physical factors (e.g., actual weather conditions), behavioral factors (e.g., adaptive behavior to restore the heat balance and previous activities) and psychological factors (e.g., thermal history and expectations) also play important roles in assessing the influence of thermal environments on human comfort (De Dear and Brager 1998a; Nikolopoulou et al. 2001; Lin 2009; Yang et al. 2013). Previous studies typically focused on citizens who share the same thermal history (Feriadi and Wong 2004; Hwang et al. 2010; Klemm et al. 2014). Knez et al. (2009) proposed a conceptual model to reveal direct and indirect effects of a given place on human thermal responses. They found that long-term memory significantly influenced people’s experience of, and expectations towards, the weather and the appreciation of outdoor urban places. People’s long-term memory on thermal comfort differs with their thermal history due to the different originated regions. Therefore, a survey study across different nationalities is required to include the variability in thermal history. Furthermore, Wang et al. (2015a, b) found that small green infrastructure (e.g., a tree grove or a single tree) in a local urban area significantly affected the microclimate and human thermal comfort. This indicates that such survey should be carried out locally.

To this end, we combined a survey on human subjective responses with simultaneous field measurements of the local microclimatic parameters in a small urban area in The Netherlands. The purpose of this study was to analyze people’s thermal comfort perception and preferences in this local area, and to specify the combined effects between thermal environmental and personal factors on their thermal perception. By means of further statistical analyses, we aimed to quantitatively relate the social survey, the field measurement data, and the role of UGI in microclimate regulation.

## Methods and materials

**Fig. 1:**
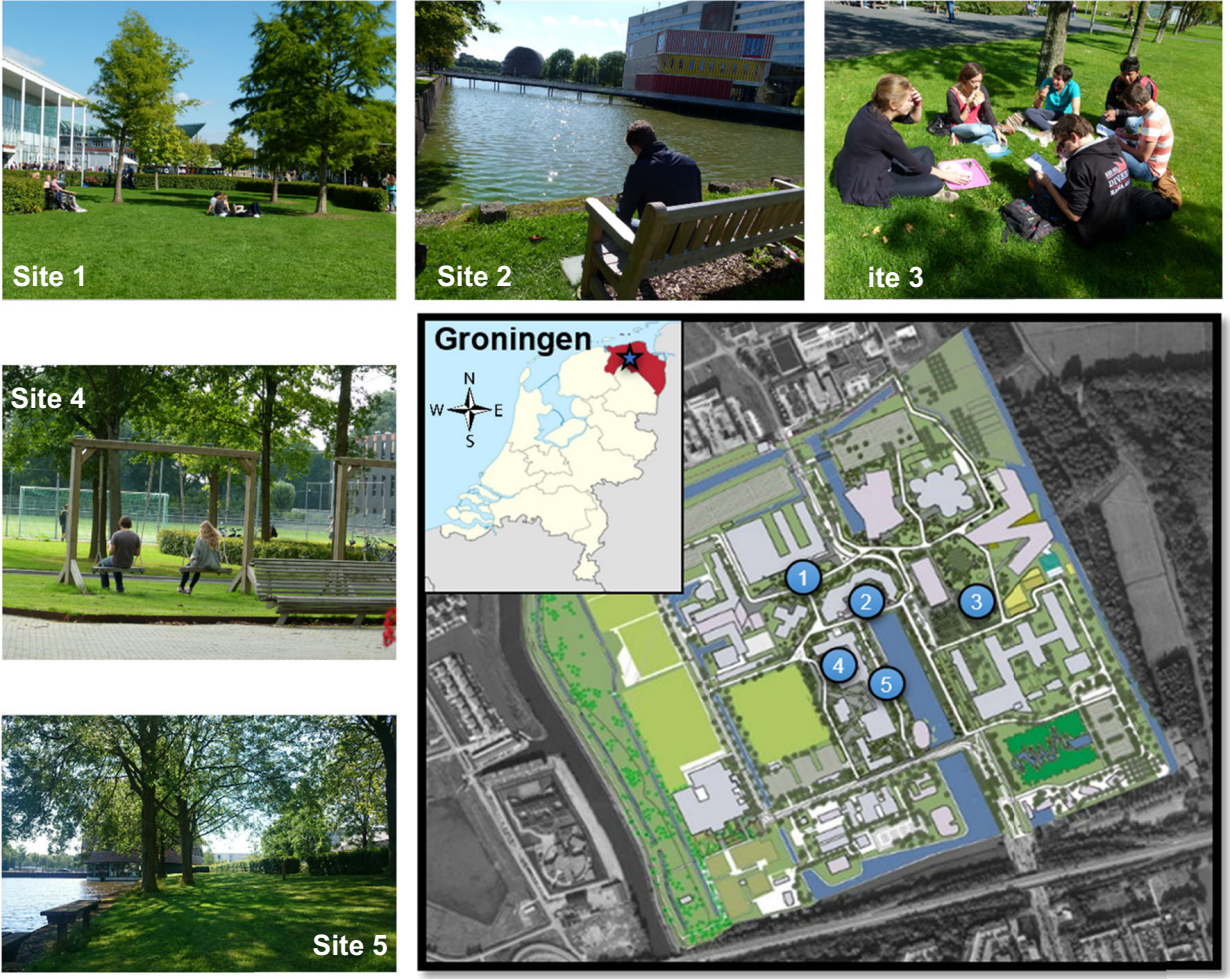
The location of the study area. The *blue star* represents the location of Groningen city. The *blue circles* represent the survey locations at the Zernike Campus, Groningen. Sources: Google Map and German Kartenwerkstatt

In this study, physical measurements of microclimatic data and a survey on people’s subjective thermal perceptions were carried out at the Zernike Campus of the University of Groningen in the northern part of The Netherlands (see Fig. 1). The information on people’s thermal perception, sensation, and preference was obtained by conducting a “right here–right now” survey among students, employees, and other people in five green urban spaces on five warm and cloudless spring and summer days in 2015. Meanwhile, mobile equipment measured air temperature (Ta), globe temperature (Tg), relative humidity (RH), and wind velocity (Va) during the survey.

### Site and field survey description

Groningen has a mild maritime climate with a moderate level of rainfall. The day lengths during summer and winter solstices are about 17 and 7½ h, respectively. Warm weather starts in April and ends in early October. The average air temperature fluctuates between 19 and 23 °C within this warm period (http://www.worldweatheronline.com). The total population of the University of Groningen is approximately 30,000 students and 5000 staff. The Zernike Campus is currently under re-construction and many “green projects” are in progress. A pilot survey with a small group of university students at the Zernike Campus was first conducted in the summer of 2014 to check if the questionnaire (see Appendix 1) was appropriate and delivered the necessary data. Afterwards, the actual survey was carried out in five green spaces with different vegetation characteristics (see Fig. 1) on five warm and cloudless days (May 11th, May 22nd, June 5th, June 12th, and July 4th 2015) from 12:00 pm to 4:00 pm. Location one was a small green space surrounded by education buildings and the second location was an open green space adjacent to water. The third and fourth locations were a small green corridor and a garden, whereas the last location was situated in a fully shaded green space by the waterside. The participants were randomly selected at the different survey locations and asked to fill out the two-page questionnaire. This questionnaire consisted of three sections:

- The first section gathered the demographical information of the respondents by asking their age, gender, nationality, weight, and body height. Additionally, to estimate the heat exchange rate, respondents’ activity level and clothing were determined according to ISO 8996 (1990) and ISO 9920 (1995) standards, respectively.
- The second section asked respondents to rate their current thermal comfort. Based on ASHRAE Standard 55 (ASHRAE 1992), a thermal sensation vote (TSV) was evaluated on a 7-point scale (−3 cold, −2 cool, −1 slightly cool, 0 neutral, 1 slightly warm, 2 warm, and 3 hot), while a Bedford 7-point scale (−3 very uncomfortable, −2 moderately uncomfortable, −1 slightly uncomfortable, 0 neutral, 1 slightly comfortable, 2 moderately comfortable, and 3 very comfortable) (Bedford 1936) was used for the thermal comfort vote (TCV). In addition, respondents were asked to indicate their thermal preference vote (TPV) on a 5-point scale ranging from “much warmer” to “much cooler.” Using the humidity sensation vote (HSV) and the wind speed sensation vote (WSV), sensation and preference for humidity and air movement were also measured on a 7-point scale (HSV, −3 very dry, −2 dry, −1 slightly dry, 0 neither dry nor humid, 1 slightly humid, 2 humid, 3 very humid; WSV, −3 very low, −2 low, −1 slightly low, 0 neither low nor high, 1 slightly high, 2 high, 3 very high). Behavioral adjustment is also an important factor for evaluating the outdoor thermal comfort. Hence, respondents were asked to select what actions they would like to take if they feel too hot in this place.
- The last section asked non-Dutch respondents to indicate their residence time in The Netherlands. Subsequently, questions on the reason of coming to the survey location, frequency of visiting, and exposure time in the selected green spaces were given to all the respondents. Additionally, we asked them to describe the previous place where they were before coming to the survey location and activities 15–20 min before coming.

Because responses for some open questions were subjective and described freely, we pragmatically categorized them into related answers to obtain as many values as possible. The responses to the *reason of coming* were grouped into environment (e.g., enjoying the nice view, fresh air, or less crowded), weather/sunshine (e.g., enjoying the sunshine, comfortable temperature, or comfortable wind flow), relaxation/rest (e.g., relax, recover from intense work/study, or break from class/work), study/work, transition (e.g., passing by or waiting for class), eat/drink, and others. The *exposure time* in the study area was divided into six categories (i.e., less than 10 min, 10–15 min, 15–20 min, 20–60 min, and more than 60 min) and the visiting frequency was categorized into rarely visit, occasionally visit, often visit, and very often visit. The *previous thermal environment* experienced 15–20 min before the survey (short-term acclimatization) was classified as outdoor and indoor, while the *previous activity* in the last 15–20 min includes resting, very light activity, light activity, and medium activity (high activity was not mentioned by the respondents).

To investigate the effect of people’s thermal history on their thermal perception, the *nationality* of the respondents was categorized under the different types of climate regions according to the Köppen climate classification (cf. Peel et al. 2007), which are tropical wet, tropical monsoon, tropical dry seasonal climate, arid, semi-arid, humid subtropical, oceanic, Mediterranean, humid continental, subarctic, tundra, ice cap, and alpine climates. The *residence time in The Netherlands* was categorized into less than 0.5 year, 0.5–1 year, 1–2 years, 2–5 years, 5–20 years, and a lifetime.

### Physical measurements

#### Measurement items

Physical measurements were conducted to collect microclimatic data at the survey locations. A mobile meteorological station equipped with a globe thermometer (Heat Index WBGT Metre 2010SD, globe diameter = 75 mm) and anemometer (MS6252B Digital Anemometer) was continuously measuring the air and globe temperatures, relative humidity, and air velocity during the survey. The measurement height was about 1.1 m above the ground surface level, corresponding to the average height of the center of gravity for adults (Mayer and Höppe 1987). All measurements were simultaneously recorded and stored with a 2-s interval. As, in general, each respondent spent approximately 5 min to fill out the questionnaire, the average values in Ta, Tg, RH, and Va during these 5 min were calculated and defined as the corresponding values for each respondent, then added to the database.

#### Mean radiant and operative temperatures

To estimate the thermal comfort, the mean radiant temperature (Tmrt) is required. Using the measurement data of Ta, Tg, RH, and Va, we calculated Tmrt based on the standardized Tmrt equation from (Eq. 1) ISO 7726 (1998).

1 $$ \mathrm{Tmrt}={\left[{\left(\mathrm{Tg}+273.15\right)}^4+\frac{3.42\times {10}^9{Va}^{0.119}}{\varepsilon {D}^{0.4}}\times \left(\mathrm{Tg}-Ta\right)\right]}^{0.25}-273.15 $$

Tg: Globe temperature (°C)
Va: Air velocity (m∙s^−1^)
Ta: Air temperature (°C)
*D*: Globe diameter (75 mm)
*ε*: Globe emissivity (normally assumed as 0.95)

The operative temperature (*T*_op_) is a metric that combines the effects of air and mean radiant temperature, and was estimated to assess the effects of microclimatic conditions only. *T*_op_ can be defined as the average of the mean radiant and ambient air temperatures at the time of interview, weighted by their respective heat transfer coefficients (ASHRAE 1992).

2 $$ {T}_{\mathrm{op}}=\frac{\left[\mathrm{Tmrt}+\left(Ta\times \sqrt{10Va}\right)\right]}{1+\sqrt{10Va}} $$

Tmrt: Mean radiant temperature (°C)
Ta: Air temperature (°C)
Va: Air velocity (m∙s^−1^)

### Thermal comfort indices

Thermal comfort indices, such as physiological equivalent temperature (PET) (Mayer and Höppe 1987), predicted mean vote (PMV) (Fanger 1972), and standard effective temperature (SET*) (Gagge et al. 1986), were used to examine the link between the thermal environment and human thermal comfort. In 1997, Matzarakis and Mayer translated PMV and PET into equivalent grade of physiological stress on human beings (Fanger 1972). However, this relationship does not consider the thermal discrepancies of seasons and climate regions. De Dear and Brager (1998b) presented a solution by estimating equations to calculate adaptive PET values for three climatic periods (cool, mild, or warm). First, a link between PET and a model of adaptive comfort was established. Second, an adaptive comfort band for 90 % acceptability was applied by adding and subtracting 2.5 °C to the comfort temperature. The upper and lower limits of adaptive comfort for 90 % acceptability were established for the three climatic periods, respectively. Finally, the association between all the models and the degree of thermal stress was made by using the limits for each period. Table 1 presents the ranges of PMV, PET, and adaptive PET during cool, mild, and warm periods for different grades of thermal perception and stress (Matzarakis and Mayer 1997; De Dear and Brager 1998b).

**Table 1:** Ranges of PMV, PET, and adaptive PET for different grades of thermal perception and physiological stress (sources—according to Matzarakis and Mayer 1997; De Dear and Brager 1998b)

PMV	PET (°C)	Adaptive PET			Thermal perception	Grade of thermal stress
		Cool period	Mild period	Warm period		
−3.5	4	4	6	8	Very cold	Extreme cold stress
					Cold	Strong cold stress
−2.5	8	8	10	12		
					Cool	Moderate cold stress
−1.5	13	13	15	17		
					Slightly cool	Slight cold stress
−0.5	18	18	20	22		
					Neutral	No thermal stress
0.5	23	23	25	27		
					Slightly warm	Slight heat stress
1.5	29	27	29	31		
					Warm	Moderate heat stress
2.5	35	34	36	37		
					Hot	Strong heat stress
3.5	41	40	42	43		
					Very hot	Extreme heat stress

The RayMan model (Matzarakis et al. 2007) was utilized for estimating the thermal comfort indices. Ta, RH, Va, and Tmrt together with other parameters that describe the heat exchange processes of the human body (personal data, clothing, and activity) were the inputs required for running RayMan. Subsequently, the simulated PMV and PET values were converted to the thermal perception and grade of thermal stress (see Table 1). Compared with PMV, PET is more intuitive and comprehensive using a widely known unit (°C). This study, therefore, used the adaptive PET model for warm climates since our survey was performed on warm and cloudless days. We compared the adaptive PET to the TSV derived from the survey to analyze if peoples’ thermal perception differed from simulated results that were calculated according to objective variables.

### Statistical analysis

First, respondents’ demographic characteristics, activity level, clothing, and physical data of outdoor climate were statistically described. Afterwards, the relationship among thermal response votes, including TSV, HSV, WSV, and TCV, was determined by applying the non-parametric Spearman correlation test, as these thermal response votes were recorded at the ordinal scale and were not normally distributed.

Subsequently, a linear regression analysis determined the relationships of the subjective TSV derived from survey versus adaptive PET and *T*_op_ derived from measurements, and to calculate the neutral temperature (comfort temperature). Because the variance of thermal sensations among individuals could be large, even in the same environment (De Dear and Brager 1998a), PET and *T*_op_ were classified into different bins with an increment of 1 and 0.5 °C, respectively. The mean thermal sensation vote (MTSV) fell into the corresponding bin. The linear regression intercept determined the neutral operative temperature.

Probit analysis was applied to calculate the preferred temperature (the temperature people stated they would prefer) based on TPV, which was divided into groups for each 0.5 °C *T*_op_ intervals. The probit regression was applied for the votes of “warmer” and “cooler” temperatures against *T*_op_. The goodness of the fit of these two probit regressions was assessed by Pearson chi-square (χ^2^) tests. The intersection point of the two regressions indicated the preferred temperature at which people did not prefer either a cooler or warmer temperature (De Dear and Fountain 1994).

Finally, since a person is not a passive recipient of its ambient thermal environment, TSV is not only explained by local microclimatic conditions. TSV is also affected by various behavioral and psychological factors (e.g., adaptive behavior, acclimatization, and habituation or expectation) that are collectively referred to as thermal adaptation. To examine the effect of thermal adaptation (including both behavioral and psychological adaptation), we investigated the impact of thermal sensation based on the responses to seven questions on behavior adjustment, purpose of coming, exposure time, visiting frequency, previous thermal environment and activity, and thermal history. The non-parametric Kruskal-Wallis *H* test was applied to evaluate the difference between the variables because TSV in this study was not normally distributed.

All the data were presented based on a 95 % confidence interval at a significance level of 0.05.

## Results

### Descriptive statistics of personal parameters and physical data

The first section of the questionnaire was about the respondents’ demographic characteristics, activity level, and clothing worn. Table 2 presents descriptive statistics of this information from the survey. In total, 389 valid questionnaires were obtained from students (70 %), employees (20 %), and other people (10 %) at five locations. The survey involved respondents from 25 countries. Those countries were categorized into the Köppen climate regions. The respondents predominantly stemmed from the oceanic climate region (i.e., The Netherlands and Western Europe).

**Table 2:** Descriptive statistics of demographic characteristics, activity level, and clothing worn

Demographic characteristics										
Gender	*N* %	Male			Female			Missing		
		205			182			2		
		52.7			46.8			0.5		
Age	*N* %	15–25	26–35		36–45	46–55		>55	Missing	
		278	75		11	19		5	1	
		71.5	19.3		2.8	4.9		1.3	0.3	
Weight	*N* %	≤50	51–60		61–70	71–80		>80	Missing	
		20	57		108	103		86	15	
		5.1	14.7		27.8	26.5		22.1	3.9	
Height	*N* %	≤160	161–170		171–180	181–190		>191	Missing	
		20	95		116	117		37	4	
		5.1	24.4		29.8	30.1		9.5	1.0	
Categorical nationality	*N* %	Tropical wet	Tropical seasonal	Arid	Humid subtropical	Oceanic	Mediterranean	Humid continental	Subarctic	Missing
		12	6	1	3	322	13	24	5	3
		3.1	1.5	0.3	0.8	82.8	3.6	6.2	1.3	0.8
Present activity level	*N* %	Reclining	Seat quiet		Standing relaxed		Light activity	Medium activity	High activity	
		26	281		39		33	9	1	
		6.7	72.2		10.0		8.5	2.3	0.3	
Clothing	*N* %	Shorts	Casual clothing		Light summer cloths		Street suit	Suit and cotton coat	Winter suit and coat	Others
		42	221		105		7	4	1	9
		10.8	56.8		27.0		1.8	1.0	0.3	2.3

The minimum, maximum, mean values, and standard deviation (SD) of Ta, Tg, RH, Va, Tmrt, *T*_op_, and PET are given in Table 3. The outdoor climate condition during the survey was hot and middle-wet with high Ta and neutral RH. The wind speed was relatively low and average Va was about 1.1 m/s (SD = 0.6 m/s). Tmrt and *T*_op_ were relatively high due to the high globe temperature. Based on these meteorological data and the physical activity and clothing of the respondents, PET was calculated by RayMan model, being 21.1–54.6 °C (from “slightly cool” to “very hot”). Comparing Ta at the survey locations with that from a weather station outside Zernike Campus, we found the green spaces at the campus were warmer by about 0.1–3.0 °C than the outside area. This phenomenon is mainly related to the high density of buildings, roads, and other infrastructures that increase the air and surface temperature (Akbari et al. 2001). Especially at location two (i.e., an open green space adjacent to water), the average Ta was 3.0 °C (SD = 0.5 °C) higher than the outside. However, Ta at location five (i.e., a fully shaded green space by the waterside) was similar to that of outside area, probably because of the high density of trees. Our previous studies (Wang et al. 2015a, b) have deeply discussed the effects of different UGIs on outdoor microclimate and human thermal conditions.

**Table 3:** Minimum, maximum, mean values, and SD of the microclimatic data

	Minimum	Maximum	Mean	SD
Air temperature (Ta, °C)	21.3	33.5	27.4	3.2
Globe temperature (Tg, °C)	22.3	40.4	32.3	3.5
Relative humidity (RH, %)	29	56	39	5
Wind speed (Va, m/s)	0.2	2.9	1.1	0.6
Mean radiant temperature (Tmrt, °C)	19.4	62.1	47.2	9.3
Operative temperature (*T*_op_, °C)	23.0	40.8	34.3	3.5
Physiologically equivalent temperature (PET, °C)	21.1	54.6	36.2	7.5

### Thermal response votes and their correlation

**Fig. 2:**
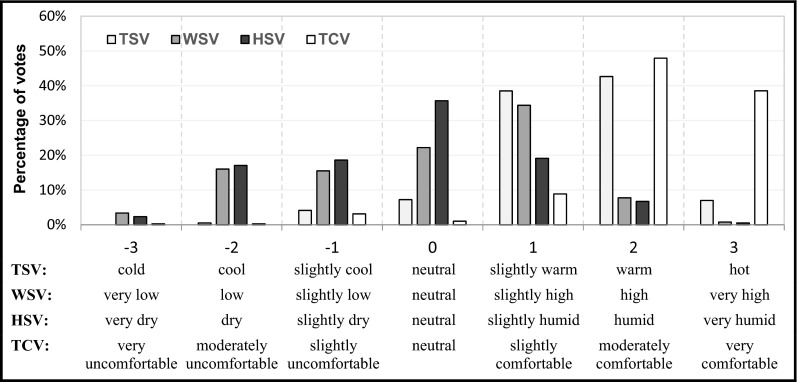
Distribution of the percentage of TSV, HSV, WSV, and TCV. *TSV* thermal sensation vote, *HSV* humidity sensation vote, *WSV* wind speed sensation vote, *TCV* thermal comfort vote

The respondents were asked to rate their instantaneous sensation of temperature, humidity, and wind. Figure 2 illustrates the percentage distribution of TSV, HSV, and WSV of all the respondents. The results showed that “slightly warm” and “warm” (+1 and +2) sensation were predominant for TSV, whereas people who felt “cool” and “cold” (−2 and −3) were rare. In terms of the humidity and wind speed sensation, people who voted “neither dry nor humid” (0) and “slightly high wind speed” (+1) represented the largest group.

The respondents’ preferences regarding the thermal, humidity, and wind speed conditions were assessed by statistically analyzing their answer to the question about their desire for “warmer/cooler,” “drier/more humid,” “less/more air movement,” or “no change” (see Appendix 2). The percentage of people who preferred “no change” in the temperature was highest (48 %), whereas the percentage of those who preferred “warmer” and “cooler” were respectively 32 and 20 %. In addition, the percentage of people who voted “no change” in humidity (69 %) and wind speed (42 %) was also higher than the other preference categories. Finally, 31 and 58 % of the respondents were unsatisfied with the current humidity and wind speed, respectively.

Table 4 shows the result of the Spearman correlation test between TSV, HSV, WSV, and TCV. Only WSV showed a significant relationship to TSV with a correlation coefficient of −0.173. This reveals that TSV tended to decrease when WSV increased. Furthermore, TCV did not show a significant relationship with TSV, HSV, and WSV. When comparing the distribution of the percentage of TSV, HSV, and WSV with TCV (see Fig. 2), people were more stringent on thermal sensation than on comfort perception. In general, around 95 % of all respondents expressed that they felt “comfortable” with all levels of comfort contained, whereas only 4 % of the respondents felt generally “uncomfortable” and 1 % voted “neutral” (see Fig. 2).

**Table 4:** Correlation analysis for thermal response votes

		TSV	HSV	WSV	TCV
TSV	Correlation coefficient	1	−0.019	−0.173 ^a^	0.056
	Sig. (2-tailed)		0.705	0.001	0.276
	*N*	389	389	389	386
HSV	Correlation coefficient	−0.019	1	0.020	−0.020
	Sig. (2-tailed)	0.705		0.689	0.689
	*N*	389	389	389	386
WSV	Correlation coefficient	−0.173^a^	0.020	1	0.013
	Sig. (2-tailed)	0.001	0.689		0.802
	*N*	389	389	389	386
TCV	Correlation coefficient	0.056	−0.020	0.013	1
	Sig. (2-tailed)	0.276	0.689	0.802	
	*N*	386	386	386	386

^a^Correlation is significant at the 0.05 level (2-tailed)

### Neutral operative temperature

As mentioned above, the PET value derived from the RayMan model was converted into the adaptive PET values for warm period. We found that the adaptive PET was mainly scored in the warm category (85 %) with the highest percentage of 25 % at “hot” thermal sensation (+3). Fifteen percent of the PET values were scored as “neutral” sensation. The ratio of PET in the cool category was very small, with <1 % at “slightly cool.” In terms of outdoor operative temperatures, the average *T*_op_ during the survey days ranged from approximately 30.5 °C on May 22nd to 40.1 °C on June 5th.

**Fig. 3:**
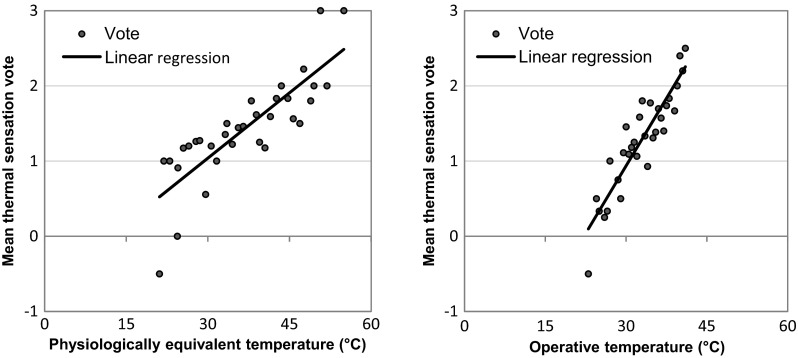
Correlation between adaptive physiologically equivalent temperature (PET) and operative temperature (*T*_op_) versus mean thermal sensation vote (MTSV)

PET and *T*_op_ were divided into a total of 33 and 31 bins with an increment of 1 and 0.5 °C, then MTSV was calculated for the corresponding bin. Adaptive PET was linearly regressed with MTSV (with 1 °C PET interval) to understand how thermal sensation varied with thermal comfort based on the energy balance of the human body. In addition, linear regression was also applied to determine the strength of the relationship between MTSV and *T*_op_. Figure 3 shows the scatter diagrams of MTSV versus PET and *T*_op_, with best-fitted lines. The simple correlation equations with 95 % confidence limits are expressed as:

3 $$ \mathrm{MTSV}=0.058\mathrm{PET} - 0.696\ \left({R}^2=0.68,p<0.0005\right) $$

4 $$ \mathrm{MTSV}=0.120{T}_{op}-2.659\left({R}^2=0.82,p<0.0005\right) $$

The values of the *t*-statistic on the coefficient of the two linear regressions were 8.224 and 11.692, respectively, while their significance level (i.e., *p* values) were both less than 0.0005 (Fig. 3). These results indicate that the variability explained by the models are robust and the coefficients are significant. From the above fitted Eq. 3, we calculated that when PET was equal to 24.5 (neutral sensation), MTSV from the survey was 0.725 (between “neutral” and “slightly warm”). Hence, people’s subjective thermal sensation was in agreement with the estimated thermal comfort. The neutrality was derived by solving Eq. 4 with MTSV equals 0; the neutral operative temperature was then calculated to be 22.2 °C.

### Preferred temperature

**Fig. 4:**
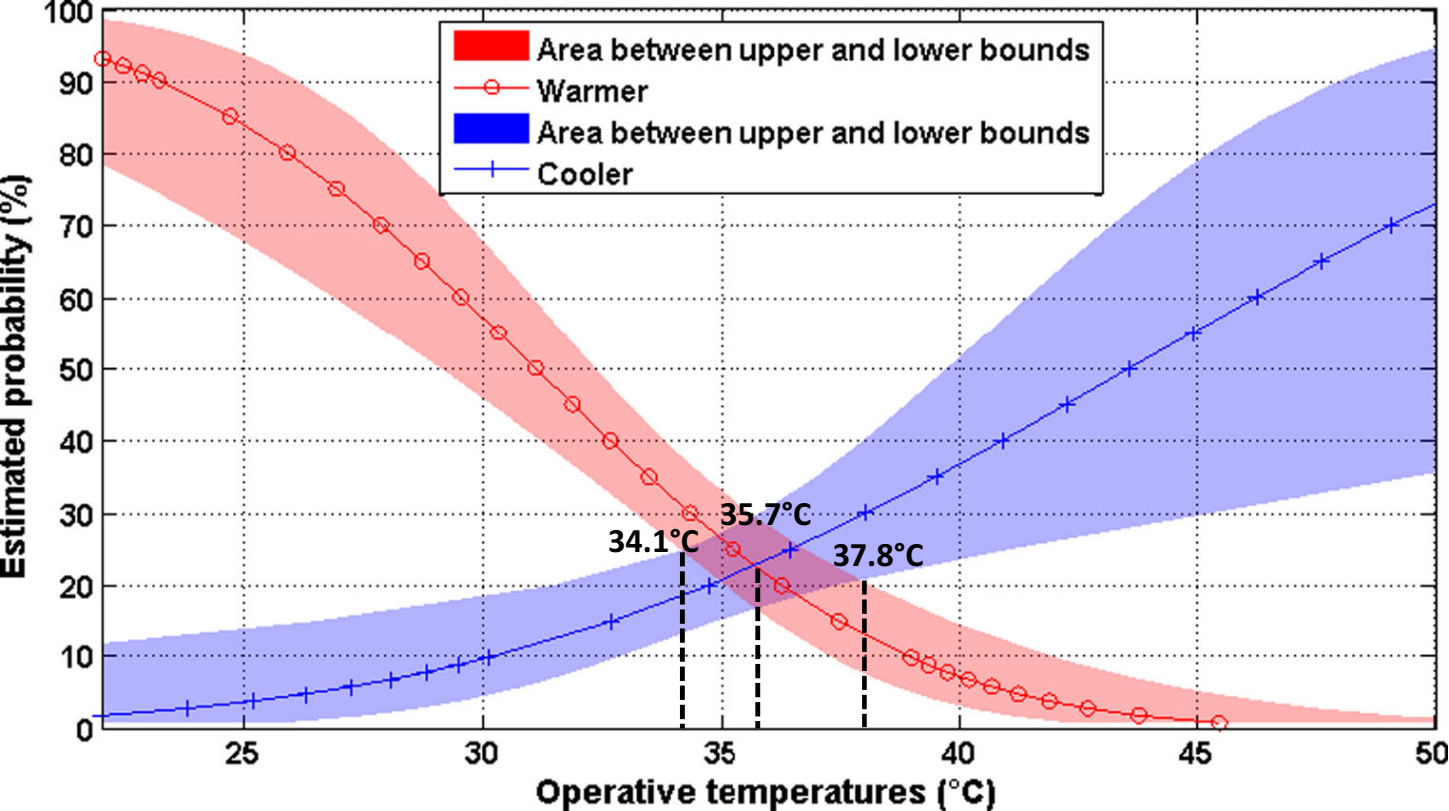
Preferred temperature based on probit analysis for “warmer” and “cooler” temperature votes against *T*_op_. *T*_*op*_ operative temperatures. Estimated probability in *y*-axis stands for percentage of respondents preferring “warmer” or “cooler”

Although the neutral operative temperature estimated using linear regression model revealed people’s comfort temperature, this temperature may not yet be equal to their actual preference. Hence, people’s TPV of “warmer” or “cooler” temperatures and the preferred temperature should also be used to define their thermal comfort perception. TPV was grouped into 31 bins for each 0.5 °C *T*_op_ intervals and fitted within the probit models for “warmer” and “cooler” temperature votes against *T*_op_. Figure 4 depicts the estimated probability values and area between upper and lower limits (95 % confidence interval) for the preference to “warmer” and “cooler” temperatures versus *T*_op_. The fits of both warmer and cooler models were good (warmer—χ^2^ = 47.033, *df* = 29, *p* = 0.018; cooler—χ^2^ = 53.752, *df* = 29, *p* = 0.003). The point at which both models intersect was assumed as the preferred temperature. This was calculated to be 35.7 °C with a range between 34.1 and 37.8 °C. Compared to the neutral operative temperature (22.2 °C) estimated by linear regression, this means an increase by more than 13.5 °C. This implies that the respondents of this study preferred much higher temperatures than the neutral operative temperature in which they already felt comfortable.

**Fig. 5:**
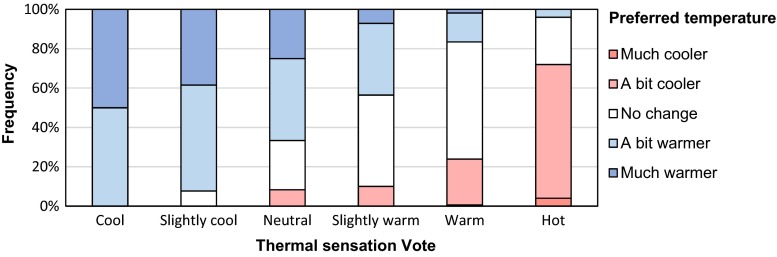
The frequencies of the preferred temperature of the respondents from temperate regions by thermal sensation vote (TSV)

Using the method mentioned above, the preferred temperatures were estimated to be 31.5 and 36.0 °C for the respondents from tropical and temperate regions, respectively. Figure 5 shows the frequencies of the preferred temperature of the respondents from temperate regions by different TSV. Generally, when TSV moves from “cool” toward “hot,” the frequencies of the preferred “warmer” temperatures declined, whereas “cooler” preference increased. However, even at warm TSV (including “slightly warm” and “warm”), considerable numbers of respondents still preferred a higher temperature (including “a bit warmer” and “much warmer”).

### Thermal adaptation

Since TSV from the survey was not normally distributed, the non-parametric Kruskal-Wallis *H* test was applied to evaluate the difference between the variables. The results are described for the following five aspects:

#### Behavioral adjustment

The respondents were asked to choose the multiple adaptive actions that they prefer to take if they feel too hot or too cold. “Move to a shaded place” was most preferred by respondents to deal with hot temperatures (48 %). Other favored adaptive actions were “get more to drink” and “reduce clothing” with 37 and 42 %, respectively. “Open umbrella/wear hat” and “nothing/go away” only occupied little percentages of 1 and 4 %. These percentages of preferred actions indicate that moving to shaded areas in outdoor spaces were more popular than using personal shading equipment or apparel. Hence, the shading provided by green infrastructure and other infrastructure in green spaces is preferred by most people to overcome their thermal discomfort.

#### Purpose of coming to the green space

The response to the open question on the respondent’s motivation to come to the survey area was grouped into seven categories (environment, weather/sunshine, study/work, relaxation/rest, transition, eat/drink, and others). The majority of the respondents (28 %) visited the green space because of the nice weather/sunshine, whereas only few people (6 %) came to enjoy the environment. The Kruskal-Wallis *H* test showed that TSV was not significantly different (*p* = 0.291) among the various purposes, indicating the reason for coming may not significantly affect thermal sensation.

#### Visiting frequency and exposure time

About 44 % of the respondents rarely or for the first time visited this green space, and 55 % of the respondents stayed more than 15 min. The results of the Kruskal-Wallis *H* test showed that the visiting frequency did not lead to significant differences in TSV (*p* = 0.242), whereas TSV was statistically different for respondents with different exposure times (*p* = 0.012). In general, TSV does not depend on the visiting frequency.

In terms of the exposure time, only 9 % of the respondents stayed in the survey area for more than 1 h, while 26, 19, 28, and 18 % stayed for less than 10, 10–15, 15–20, and 20–60 min, respectively. The longer the exposure time was, the higher the average TSV. The average TSV in the category of more than 1 h was the highest among the different exposure times.

#### Previous thermal environment and activity

**Fig. 6:**
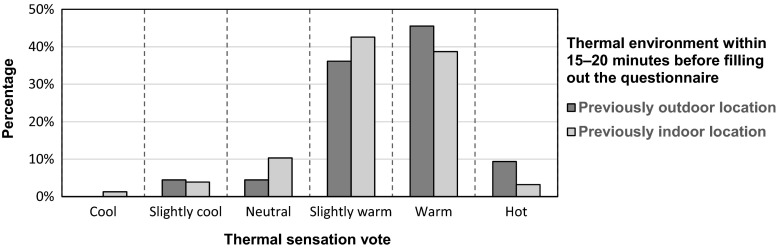
Comparison upon the percentage of thermal sensation vote (TSV) by the respondents who stayed outdoor or indoor in the last 15–20 min before filling out the questionnaire

About 41 % of respondents changed their environment from indoor to outdoor within 15–20 min before filling out the questionnaire. Based on the Kruskal-Wallis *H* test, the difference in TSV between either staying outdoor or indoor in the last 15–20 min was significant (*p* = 0.003). Figure 6 shows the percentage distributions of TSV by the respondents who stayed outdoor or indoor in the last 15–20 min. The respondents, who had been in outdoor condition before the survey, tended to choose a higher TSV compared to those who had been indoor. In addition, the Kruskal-Wallis *H* test showed that respondents’ previous activity level led to significant differences in TSV (*p* = 0.031). The average TSV was 1.52, 1.45, 1.20, and 1.08 (on a scale of 0 “neutral” to 2 “slightly warm”) for resting, very light activity, light activity, and medium activity, respectively. In other words, a lower previous activity level resulted in higher average TSV. We also found that people who were previously resting stayed longer in the green space, while those who were previously active had stayed shorter. Hence, the differences in average TSV could be a result of synergism between previous activity and exposure time.

#### Thermal history

As mentioned earlier, people’s thermal history could affect their expectations of thermal conditions in the survey area. The Kruskal-Wallis *H* test was first performed to evaluate the difference of TSV by the respondents from different regions with various types of climate. We found that TSV was statistically different (*p* = 0.041) for respondents with a different thermal history. The average TSV of the respondents from tropical countries was the lowest (0.8, SD = 1.1), while respondents from subarctic countries showed the highest average TSV (1.8, SD = 0.8). In addition, we found that respondents from temperate regions preferred higher temperatures compared to those from tropical regions, although the climate in the home country did not significantly affect respondents’ preferred temperature (*p* > 0.05). This suggests that people’s thermal history could affect their thermal sensation and expectation in outdoor spaces. However, the distribution of the subject samples was quite unequal and concentrated in European countries (i.e., temperate regions). The sample sizes of the other climate groups, especially the cold climate, were relatively small to permit formal comparison. This statistical relationship is, therefore, probably biased.

Furthermore, the respondents were also asked how long they have been in The Netherlands. The statistical test results showed that TSV was not significantly influenced by the residence time in The Netherlands, with *p* = 0.776.

## Discussion

### Correlation between thermal responses votes

The result of the Spearman correlation test confirmed the significant relationship between WSV and TSV with a correlation coefficient of −0.173. The finding was generally in accordance with previous studies that reported that the increase of WSV significantly decreased TSV with a correlation coefficient of −0.03 to −0.78 (Givoni et al. 2003; Cheng and Ng 2008; Nikolopoulou 2004; Krüger et al. 2011; Yang et al. 2013). No significant relationship between TSV and HSV was found in our study. This finding contrasts with previous studies (Nikolopoulou 2004; Yang et al. 2013) that showed that HSV had a significant effect on TSV (but with a quite different correlation coefficient from −0.09 to 0.01). In fact, the influence of humidity on thermal comfort is likely to be different depending on the range and value of humidity. The range of RH in our study (29–56 %) was smaller than that was reported by Yang et al. (2013) (48–91 %) and Nikolopoulou (2004) (20–80 %), and the maximum RH was also much smaller. Although respondents subjectively voted HSV as “dry” and “humid,” the absolute RH value was too low to significantly influence their TSV.

Furthermore, TCV did not show a significant relationship with TSV, HSV, and WSV. People were more stringent on thermal sensation than on comfort perception since 95 % of the respondents expressed that they felt “comfortable,” but only 7, 22, and 36 % of the respondents voted “neutral” for thermal, humidity, and wind speed sensations, respectively. This indicates that they preferred a change in thermal condition, but were satisfied with the ambient environment in the green space. It appears that people’s assessment of their comfort is not only based on the current thermal condition in green spaces. Other environmental and non-physical factors, such as natural view, quiet environment, and emotional condition, also affect people’s comfort assessment (Givoni et al. 2003; Feriadi and Wong 2004). In addition, this study performed surveys at five locations over the 5 days to involve respondents with different backgrounds as much as possible. The different environmental conditions possibly affected people’s impressions regarding usage of that space and change their comfort perception. Moreover, the environmental condition may also influence people’s feeling about the level of warmth of the environment (Rohles 2007). Applying Kruskal-Wallis *H* test to evaluate the difference of TSV among the survey locations at each *T*_op_ bin, we found that the variation of location did not lead to significantly different TSV values at all *T*_op_ bins. Hence, the influence of the different environmental conditions of five survey locations on people’s thermal sensation was negligible in this study.

### Neutral operative and preferred temperature

The neutral operative temperature and preferred temperature were assessed using linear regression and probit models based on the data from the survey (TSV and TPV) and corresponding measurements (*T*_op_). The outcome of the analysis indicated that the neutral operative temperature of 22.2 °C had been interpreted by respondents as an acceptable temperature. This result is generally in line with previous studies carried out in other temperate European regions which reported a neutral operative temperature of approximately 21.5 °C (e.g., Nikolopoulou and Lykoudis 2006). Our neutral operative temperature is lower than those reported by studies in tropical regions: 26.5–27.9 °C in Taiwan (Hwang et al. 2010) and 28.7 °C in Singapore (Yang et al. 2013). Interestingly, based on our analysis, the respondents subjectively preferred a much higher operative temperature (35.7 °C, ranging 34.1–37.8 °C in its 95 % confidence interval) compared to their neutral operative temperature. This preferred temperature strongly differed from the previous studies (Brager and de Dear 1998; Lin et al. 2011; Yang et al. 2013; Hwang and Lin 2016), who reported a preferred temperature of 25–29 °C. Yang et al. (2013) concluded that people in hot and humid climates dislike describing their preferred state as “warm” because that word implies an undesirable state. This conclusion could explain the extremely high preferred temperature derived in this study. Applying the probit analysis for the respondents from tropical and temperate regions respectively, we found the respondents from temperate regions preferred higher temperature (36 °C) than those from tropical regions (31.5 °C). In this study, most respondents come from temperate regions with a relatively cool climate. These respondents, who rated their current thermal condition as “slightly warm,” still preferred “a bit warmer” (36 %) and “much warmer” (7 %). We therefore conclude that people from temperate regions instinctively like to describe their preferred state as “warmer” instead of “cooler,” even if they already feel warm. In addition, relatively short exposure time in this study might be another reason why people preferred warmer temperature. The exposure time had a negative effect on people’s preferred temperature (*p* = 0.003). The high preferred temperature may be due to a preponderance of people staying for relatively short time at the survey locations (91 % of the respondents stayed in the survey area for less than 1 h).

### Thermal adaptation

The Kruskal-Wallis *H* test of subjective TSV and thermal adaptation confirms the effect of exposure time, previous thermal environment, and activity on thermal comfort. People who are engaged in high activities 15 to 20 min before the survey expressed a cooler thermal sensation when filling the questionnaire than those with lower or no activity. A plausible explanation is that people with a relatively high previous activity might feel less hot due to their warmer body temperature. On the other hand, we found that people with a lower previous activity level had longer exposure time in the green space, resulting in non-causality between previous activity and TSV.

Additionally, people from hot regions generally expressed a relatively cooler thermal sensation (“slightly warm”) than those from cold regions who chose relatively warmer thermal sensation (“warm”) under similar conditions. That people who live in hot regions are more tolerant to hot conditions would be a logical explanation. Although the climate in the respondents’ home country did not significantly affect their preferred temperature, respondents from tropical regions commonly preferred “cooler” temperatures, while those from temperate regions preferred “warmer” temperatures. This result is in line with the preferred temperatures derived from probit models, meaning that people’s thermal experience and history influenced their preferred state and led them to prefer warmer or cooler temperatures. Of course, this does not mean that UGIs have a negative influence on people’s thermal comfort in cold regions, as *T*_op_ in the summer could be much higher than respondents’ comfort and preferred temperature. In addition, UGIs are also desirable from a wind shelter or aesthetic point of view. Nevertheless, partly shaded areas might be preferred above totally shaded areas in cold regions. Hence, the planning and management of UGIs should take account of people’s thermal preferences in different regions. Notably, our sample sizes of the climate groups were quite unequal and concentrated in European countries, indicating this statistical relationship is probably biased. To make this result more robust, future studies should involve more international participants who are from outside Europe.

Moreover, the exposure time was found to have a significant impact on the TSV. The longer the exposure time was, the higher the TSV value. However, all the categories of exposure times in this study were relatively short, i.e., the longest exposure time was more than 1 h. A longer exposure time may enhance people’s tolerance to hot conditions and lead to a different result.

## Conclusions

This study analyzed people’s thermal comfort perception and preference in a local area, specified the combined effects between thermal environmental and personal factors on people’s thermal comfort, and established a quantitative relationship between the combined use of a social survey and field measurements to determine the role of UGI in microclimate regulation.

The data collected from surveys and measurements at the Zernike Campus of University of Groningen provide important information on how people perceive thermal comfort in local green spaces. Samples were randomly drawn from a group consisting of different nationalities. This allowed us to examine the influence of people’s thermal history on their thermal sensation and expectation. However, the participants were mainly from European countries, and the samples from the other climate regions were relatively small. Hence, the statistical relationship between the climate regions and TSV may be biased and requires a larger sample size from outside Europe. In addition, we concluded that non-physical environmental and subjective factors (e.g., natural view, quiet environment, and emotional background) played more important roles in the comfort perception than the actual thermal conditions.

The subjective thermal sensation from the survey was in agreement with the estimated thermal comfort based on the measurements, and the comfort temperature was estimated to be 22.2 °C. However, we found a considerably higher preferred temperature (i.e., 35.7 °C) especially expressed by people from temperate regions. The Kruskal-Wallis *H* test showed the effect of the previous thermal environment and activity experienced immediately prior to the survey and the influence of long-term thermal history on human thermal comfort. Although the effect of long-term thermal history needs further investigation, including people’s thermal preferences and adaptation factors is necessary when interpreting results from human thermal comfort research in urban green spaces.

The combined use of a “right here–right now” survey and simultaneous measurements of weather conditions is essential to understand and quantify the combined effects of objective thermal environmental factors and subjective personal perception on people’s thermal comfort. By providing evidence for the impacts of both objective and subjective factors on human thermal comfort, the relationship between UGI, microclimate, and thermal comfort that was specified in this study can assist urban planning to make better use of green spaces for microclimate regulation.

## Figures and Tables

**ᅟ:**
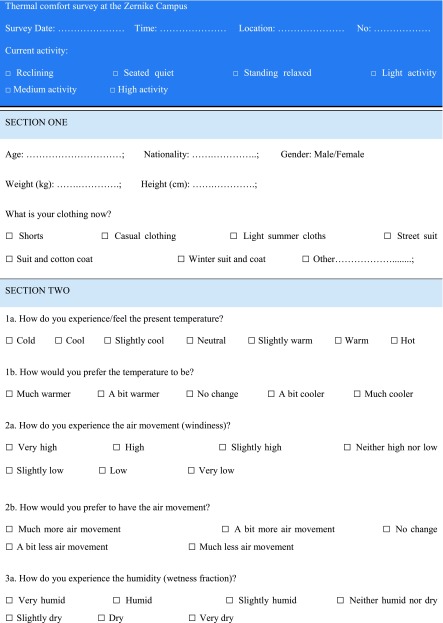

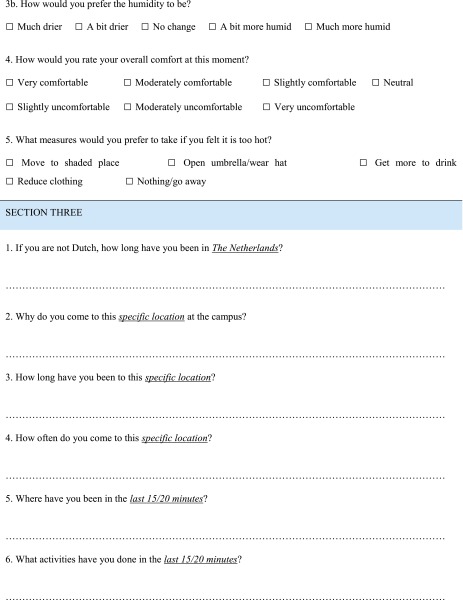
The questionnaire for the thermal comfort survey at the Zernike Campus

## Appendix 2

The respondents’ preferences regarding the thermal, humidity, and wind speed condition

**Table:**

Respondents’ preferences	−2		−1		0		1		2	
	*N*	%	*N*	%	*N*	%	*N*	%	*N*	%
Thermal	Much cooler		A bit cooler		No change		A bit warmer		Much warmer	
	2	0.5	77	19.8	186	47.8	99	25.4	25	6.4
Humidity	Much drier		A bit drier		No change		A bit more humid		Much more humid	
	9	2.3	58	14.9	268	69.1	52	13.4	1	0.3
Wind speed	Much less air movement		A bit less air movement		No change		A bit more air movement		Much more air movement	
	41	10.5	98	25.2	163	41.9	77	19.8	10	2.6
